# The Effect of Tensile Hysteresis and Contact Resistance on the Performance of Strain-Resistant Elastic-Conductive Webbing

**DOI:** 10.3390/s110201693

**Published:** 2011-01-28

**Authors:** Tien-Wei Shyr, Jing-Wen Shie, Yan-Er Jhuang

**Affiliations:** Department of Fiber and Composite Materials, Feng Chia University, 100 Wenhwa Road, Taichung 40724, Taiwan; E-Mails: P9521309@fcu.edu.tw (J.-W.S.); elaine052773@hotmail.com (Y.-E.J.)

**Keywords:** elastic-conductive webbing, textile strain-resistant sensor, tensile hysteresis, contact resistance

## Abstract

To use e-textiles as a strain-resistance sensor they need to be both elastic and conductive. Three kinds of elastic-conductive webbings, including flat, tubular, and belt webbings, made of Lycra fiber and carbon coated polyamide fiber, were used in this study. The strain-resistance properties of the webbings were evaluated in stretch-recovery tests and measured within 30% strain. It was found that tensile hysteresis and contact resistance significantly influence the tensile elasticity and the resistance sensitivity of the webbings. The results showed that the webbing structure definitely contributes to the tensile hysteresis and contact resistance. The smaller the friction is among the yarns in the belt webbing, the smaller the tensile hysteresis loss. However the close proximity of the conductive yarns in flat and tubular webbings results in a lower contact resistance.

## 1. Introduction

Electronic textiles (e-textiles) can be used in the entertainment industry, fashion industry, communications, as well as for sensing, monitoring, and even locating applications [1–6]. The advantages of e-textiles are not only that they are light, flexible, durable, provide ventilation, and are easily formed, but they are also electrically conductive. One particularly interesting application for e-textiles is its use as a strain-resistance sensor. Changing the resistance of a conductor by stretching was first reported by Lord Kelvin [7]. However, because of the rigidity of the gauges, the maximum static strain level that can be applied before failure the measurements were limited to low stress measurements only. Fatigue is another problem in dynamic measurement because of the poor repeatability of alloys in stretch-recovery cycles [7].

Elasticity and conductivity are the two main requirements for e-textiles as a strain-resistance sensor. Tao studied a series of large-strain gauges, in which elasticity was obtained by a knitted structure or by Lycra fiber [7–12]. Abdessalem reported that plated plain knitted fabric using Lycra yarn exhibited serious tensile hysteresis [13]. The recovery in knitted fabric is incomplete depending on the proportion of Lycra yarn in the fabric. This partial non-recovery of knitted fabric is due to the hysteresis of spun yarns having a plastic deformation behavior, which is linked to slippage of the cotton fiber and viscoelasticity. Wu studied polypyrrole (PPy)-coated nylon Lycra fabric [14]. He found that the resistance of PPy-coated nylon Lycra fabric decreased when stretched, but that the tensile hysteresis was significant due to changes in the structure. Strain-resistance sensors, with conductive material coated on the textiles, easily form cracks in the conductive layer when being stretched. This results in poor linearity and repeatability of the relationship between resistance and strain [15]. Sensors based on conductive polymer composite composed of thermoplastic elastomer filled with black particles were studied [16,17]. Mattmann developed a strain sensor using a mixture of thermoplastic elastomer (TPE) and carbon black particles [18]. It proved to have a linear resistance response to strain, but with a small electrical hysteresis, with a maximum hysteresis error ±3.5% (7%) in the strain response.

The use of carbon coated yarns wrapped with elastic yarn as a strain sensor was studied by Huang [19,20]. It was found that a soft-core yarn sensor can achieve high resistance sensitivity with low linearity. When the sensor consists of high-density piezoresistive fibers, it can achieve high linearity with low resistance sensitivity. The non-linearity of the sensor in the strain-resistance relationship was mainly due to the irregular characteristic of the yarn structure [19,20]. Tao observed the change in contact resistance on the textile strain sensor [8]. The contact resistance between two contacting yarns dominates the sensing performance. The contact points of the carbonized single warp knitted fabric influenced the sensitivity, repeatability, hysteresis, linearity, and strain range of the sensor [8]. The hysteresis is caused by friction and structural changes in the fabric. The hysteresis increases with the decrease of fabric density, which determines the number of contact points within a given length of fabric.

Hu found that a higher level (but close to the percolation threshold) of carbon nanotubes in a polymer composite would increase the resistance sensitivity of that composite [21]. Berger reported that access to a semiconductor region by means of a metal contact usually exhibits a higher resistance than expected from an ideal contact [22]. Komvopoulos reported that although the contact surfaces of microdevices consist of structural polysilicon layers surfaced with gold, the presence of contaminants and insulating films between the contact interfaces may greatly increase the electrical contact resistance [23]. Tersoff reported that if conduction requires either scattering or tunneling, the resistance can easily become much larger [24]. Liu reported that the contact resistance is associated with the conduction characteristic of the contact surface [25]. Slade calculated the electrical contact resistance as the sum of the constriction resistance and the interfacial film resistance [26].

However, the structure of a textile, the property of high tensile elasticity with low hysteresis and the property of high electrical resistance sensitivity with low contact resistance in the stretch-recovery cycles are of greater interest as a strain-resistance sensor. In this paper, we designed three kinds of webbings, including flat, tubular, and belt ones, made with Lycra fiber and carbon coated polyamide fiber, which were used as strain sensors. The strain-resistance properties in the stretch-recovery measurement of elastic-conductive webbings were investigated. The effect of the webbing structures on the tensile hysteresis and the electrical contact resistance is analyzed and observed experimentally. The electrical resistance sensitivity of each of these webbings was measured as well.

## 2. Experimental Section

In this study three kinds of webbing structures including flat, tubular, and belt webbings were constructed using conductive yarns and elastic yarns. Polyamide fiber coated with carbon particles (PAC fiber) was used as the conductive fiber (diameter of 50 μm). Fifteen PAC fibers were twisted with a bulky polyester yarn to form a conductive yarn (diameter of 420 μm, 329 kÙ/10 cm). The number of twists per meter of the conductive yarn was 80. Lycra fiber was cross-wrapped by applying two polyester yarns to form an elastic yarn (diameter of 800 μm).

**Figure 1.**
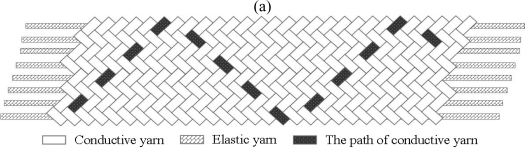

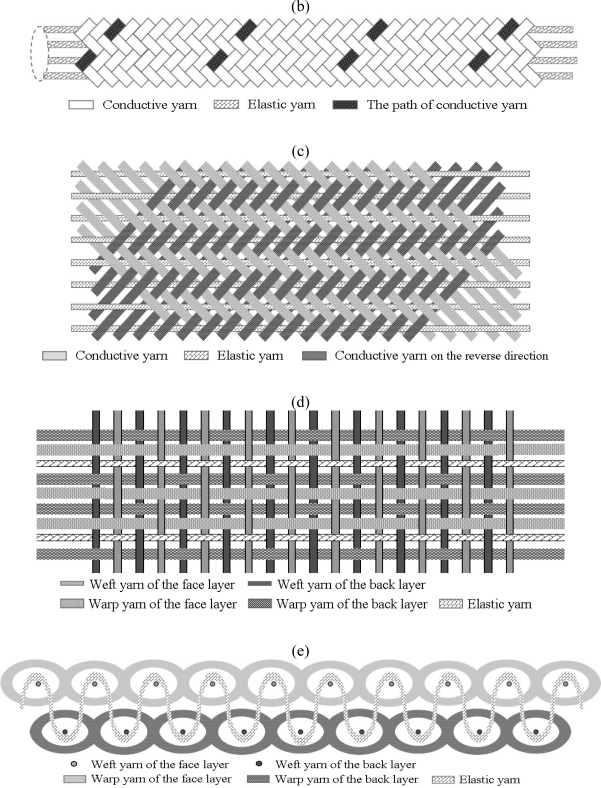
Structure of **(a)** flat webbing, **(b)** tubular webbing, **(c)** the laid-in elastic yarns of the flat and tubular webbings, **(d)** belt webbing, and **(e)** elastic yarn traveling back and faced the layers.

The schematic webbing structures are shown in Figure 1. Flat and tubular webbings were plaited by conductive yarns in a diagonal pattern over and under two yarns [see Figure 1(a,b)]. The elastic yarns were positioned between the conductive yarns as stuffer yarns in the webbing direction [see Figure 1(c)]. Belt webbing consisted of two separate layers with a plain pattern structure, with the warp yarns and weft yarns being interlaced at right angles [see Figure 1(d)]. The elastic yarns were laid in the warp direction of the belt webbing, while the elastic yarns acted as connecting yarns traveling back and faced the layers to hold them together [see Figure 1(e)]. The weft yarn (diameter of 130 μm, 3.6 MÙ/10 cm) was made from six PAC fibers twisted with one polyester yarn, and the warp yarn was a conductive yarn. The number of elastic yarns and conductive yarns, the density of the weft yarn, and the feed ratio of the conductive yarns for the three webbings are listed in Table 1. The characters E, C, and D in the webbing code indicate elastic yarn, conductive yarn, and the density of weft yarn, respectively. In the present paper the feed ratio difference of the conductive yarns among the webbings resulted in the structural stability limitation of the webbings.

**Figure 2.**
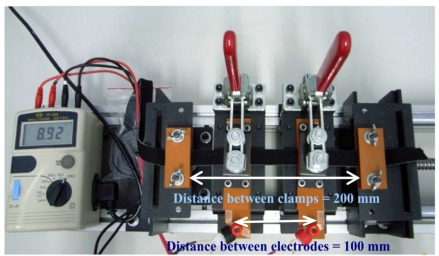
A self-assembled apparatus for measuring the strain-resistance.

The tensile property of the elastic-conductive webbings was measured using a servo control universal testing machine (GT-7001-MC01). The clamping distance was set at 200 mm. Each sample was measured in ten stretch-recovery cycles with 30% strain. The stretch-recovery speed was a constant 15 mm/s. The resistance of the elastic-conductive webbings was evaluated for the strain-resistance measurement using a self-assembled apparatus with a milli-Ohm meter (YF-508). The clamping distance was set at 200 mm, and the distance between electrodes was set at 100 mm. A pair of copper electrodes with contact length of 1 cm was used (see Figure 2). The strain-resistance of the sample was measured by the stretch-recovery tests within 30% strain. All of the samples had been subjected to a pre-stretch-recovery cycle prior to the measurement. After the measurements the data were fitted by a linear model.

## 3. Results and Discussion

### 3.1. Tensile Hysteresis of Webbings

**Figure 3.**
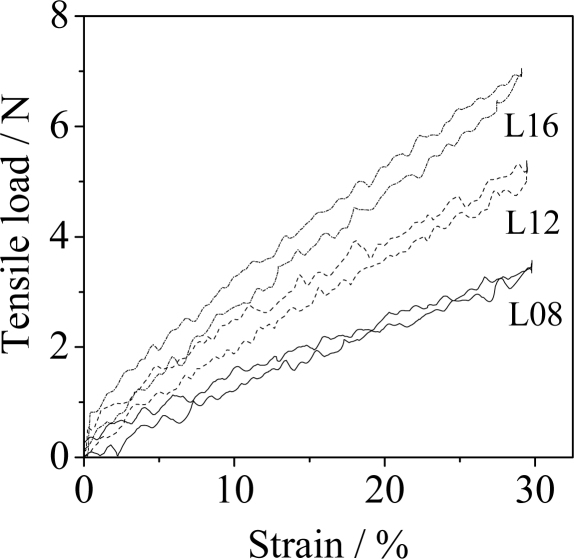
Hysteresis loops for 8, 12, and 16 Lycra fibers in the stretch-recovery cycle at 30% strain.

The typical stretch-recovery cycle of Lycra fibers using a variety of number of strands are shown in Figure 3. The tensile load was proportional to 30% strain, with coefficients of determination (R^2^) in the range of 0.98∼0.99, for the stretching and the recovery curves of the samples. Here, the coefficient of determination (R^2^) is such that 0 < R^2^ < 1, and represents the percent of the data that is the closest to the line of best fit [27]. However, in the stretch-recovery measurement the hysteresis phenomenon of the Lycra fiber was obvious. This was due to the friction and structural changes in the molecular chains of the Lycra fiber during stretching and recovery. The hysteresis loss is defined as the area of the hysteresis loop, which is the area between the two curves-stretch and recovery-represents the work loss to the webbing. Although the hysteresis loss increased with the increase in the number of Lycra fibers (see Table 2), the ratio of the hysteresis of the Lycra fibers (H_L_) to the work, the area under the load-extension curve, at 30% strain decreased from 11.6% for eight Lycra fibers (L08) to 9.6% for sixteen Lycra fibers (L16). The tensile load of elastic yarn showed a good linear relationship to the strain within 30%, with the coefficients of determination (R^2^) being in the range of 0.94∼0.99, for the stretch and the recovery curves of the samples. However the hysteresis loss of the elastic yarn was greater than that of Lycra fiber (see Table 2). In the present study the hysteresis loss of the samples (H) was defined as the sum of the hysteresis loss of the Lycra fiber themselves (H_L_) and the friction among the fibers (H_F_). The ratio of H_F_ to H in the elastic yarns still remains at 15.2% for the eight elastic yarns (E08). The friction effect between the Lycra fiber and the cross-wrapped polyester yarns is obvious.

**Figure 4.**
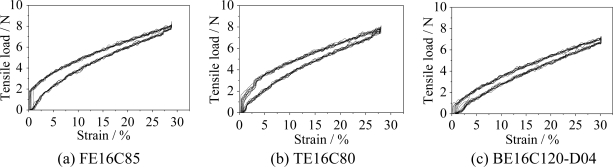
Typical ten stretch-recovery cycles with 30% strain of **(a)** flat, **(b)** tubular, and **(c)** belt webbings using sixteen elastic yarns.

Figure 4 shows the typical ten stretch-recovery cycles at 30% strain of three webbings. All the webbings had good tensile linearity in the stretch and the recovery curves within 30% strain. The tensile load was proportional to the strain within 30%, with coefficients of determination (R^2^) being in the range of 0.90∼0.99, for all of the samples. The tensile loads in the different webbings were close to the same at 30% strain. Based on the tensile performance of the Lycra fibers in Figure 3, it is evident that the tensile load of the webbings mainly contributed on the Lycra fibers.

The tensile hysteresis loss of webbings is more than that of the corresponding elastic yarns (see Table 2). The flat and tubular webbings have a similar webbing structure, and are plaited with two conductive yarns in a diagonal pattern with laid-in elastic yarns. The hysteresis losses of flat and tubular webbings show no significant differences (see Table 2). In the belt webbing two separated layers were connected by elastic yarns. The friction among these yarns was less than that in flat or tubular webbings. Thus, the hysteresis loss of flat and tubular webbings is higher than that of belt webbing when they have the same elastic yarns. The tensile hysteresis loss of BE08C56-D04 webbing was 17.6 ± 0.7 N·mm in the stretch-recovery measurement at 30% strain. The ratio of H_F_ to H in BE08C56-D04 webbing approaches 23.9%. When the number of elastic yarns in the belt webbing increased to sixteen, the ratio of H_F_ to H in BE16C120-D04 webbing increased to 34.9%. The ratio of H_F_ to H in flat and tubular webbings shows the same trend. The increase of the number of yarns in the webbing results in an increase in friction among the fibers within the webbings and increases the loss of hysteresis of the webbing. When the weft yarn density of the belt webbings increases the friction between the warp yarns and the weft yarns increases (see Table 2). This results in an increased loss of tensile hysteresis in the belt webbing. The ratio of H_F_ to H in BE12C88-D12 webbing increased to 66.5%. It is evident that the hysteresis loss of the webbings in the stretch-recovery measurement within 30% strain is a result of the Lycra fiber itself, the friction among the yarns, and the webbing structures. The number of Lycra fibers, the yarn number, and the webbing structure significantly affect the loss of hysteresis of the webbings in the stretch-recovery measurement.

### 3.2. Contact Resistance of Webbings

The measured resistance of the received PAC fiber, the conductive yarn, and the webbings are shown in Table 3. The results show that the less the number of PAC fibers and the higher the feed ratio of the conductive yarn, the higher the resistance of the webbing will be. The normalized resistance of the PAC fibers (R_ny_) in the conductive yarn was calculated as follows: R_ny_ = R_my_ × N_y_. Here, R_my_ is the measured resistance of the conductive yarn per 10 cm and N_y_ is the number of the PAC fibers in the conductive yarn. The normalized resistance of the PAC fibers (R_nw_) in the webbing was calculated as follows: R_nw_ = R_mw_ × N_w_ ÷ L_w_. Here, R_mw_ is the measured resistance of the webbing per 10 cm, N_w_ is the number of the PAC fibers in the webbing, and L_w_ is the feed ratio of conductive yarn in the webbing (see Table 3). The results show that the normalized resistance of the PAC fiber per 10 cm increased in the order of the received PAC fiber, followed by conductive yarn, and than the webbings. This is due to the fact that the contact resistance of the samples, which is defined as the ratio of the voltage across the contact to the current flowing through a closed pair of contacts. The contact resistance is associated with the conduction characteristic of the contact surface. The larger the contact area and the less the impurity of the pair materials surface is, the better the conductivity and the lower the resistance [20].

The fifteen PAC fibers were twisted to form a circular conductive yarn. The current flows across the PAC fibers through a pair of electrodes to measure the resistance of the conductive yarn. The contact resistance was created as a result of the conduction characteristic of the contact surfaces of the PAC fibers and the conductive yarns in the webbing, as well as the conduction characteristic of the contact surfaces between the webbing and the pair of electrodes. The normalized contact resistance of the PAC fiber per 10 cm of conductive yarn and webbings are given in Table 4. The contact resistance (R_c_) was calculated by subtracting the ideal resistance (R_i_) from the measured resistance (R). The normalized contact resistance of the PAC fiber (R_ncy_) in conductive yarn was calculated as follows: R_ncy_ = R_cy_ × N_y_. Here, R_cy_ is the contact resistance of conductive yarn per 10 cm. The contact resistance of conductive yarn (R_cy_) was calculated as follows: R_cy_ = R_my_ − R_iy_. Here, R_iy_ is the ideal resistance of the conductive yarn per 10 cm. R_iy_ was calculated as follows: R_iy_ = 4,077 ÷ N_y_. In the study the measured resistance of the received PAC fiber (4,077 kΩ) was used as the ideal resistance. The normalized contact resistance of the PAC fiber (R_ncw_) in webbing was calculated as follows: R_ncw_ = R_cw_ × N_w_ ÷ L_w_. Here, R_cw_ is the contact resistance of webbing per 10 cm. The contact resistance of webbing (R_cw_) was calculated as follows: R_cw_ = R_mw_ − R_iw_. Here, R_iw_ is the ideal resistance of webbing per 10 cm. R_iw_ was calculated as follows: R_iw_ = 4,077 ÷ N_w_ × L_w_. It is evident that the amount of contact resistance in the conductive yarn is significant. The ratio of contact resistance to measured resistance in the conductive yarn was close to 17.3%.

When the conductive yarns were plaited in flat and tubular webbings or interlaced into belt webbings, the ratio of the contact resistance to the measured resistance of the webbing increased. Compared with the conductive yarns in the flat and tubular webbings, the conductive yarns in the warp of the belt webbing were separated by the weft yarns. The contact resistance in the belt webbing is higher than in the flat and tubular webbings due to the contact characteristic between the conductive yarns and the electrodes. The ratio of contact resistance to measured resistance of the flat, tubular, and belt (BE12C88-D04) webbings are 20.8%, 21.1%, and 26.5%, respectively. The normalized contact resistance of the PAC fibers per 10 cm increased in the order of conductive yarn, followed by tubular and flat webbings, and then the belt webbing (see Table 4).

When the weft yarn density increased in the belt webbing, the contact characteristic between the warp yarns and the electrode were different. The higher the weft yarn density, the more it obstructs the contact between the warp conductive yarns and the electrodes. The contact resistance of the belt webbings increased in the order of BE12C88-D04, BE12C88-D08, and then BE12C88-D12. Here the ratio of the contact resistance to the measured resistance of the BE12C88-D12 webbing reaches 31.1%. The effect of the weft yarn on the contact resistance is significant. The more weft yarn there is in the belt webbing, the more contact resistance there is in the belt. The contact resistance of webbings is not only influenced by the number of conductive yarns, but also by the webbing structure and the weft yarn density.

Due to the presence of contact resistance in the samples, the normalized resistance of the PAC fibers per 10 cm decreases in the order of belt webbing, flat and tubular webbings, conductive yarn, and the received PAC fiber (see Table 3). The present study shows no significant difference in the normalized resistance of the PAC fibers per 10 cm between flat and tubular webbings. In belt webbings, the normalized resistance of PAC fiber per 10 cm increases with the increase of weft yarn density. The better the contact between the conductive yarns in the warp and the electrodes, the lower the contact resistance in the webbing. The measured resistance of the webbing is not only affected by the number and the feed ratio of the conductive yarns, but also by the contact resistance of the webbing.

### 3.3. Resistance Sensitivity of Webbings

**Figure 5.**
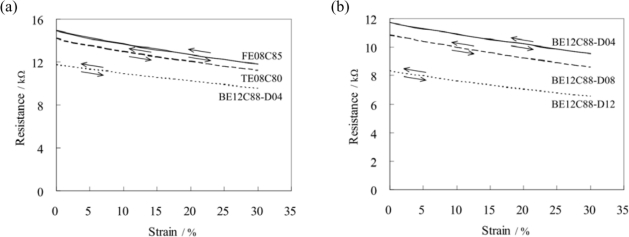
Relationship between the resistance and the strain in the stretch-recovery curves **(a)** varied webbing structures and **(b)** weft yarn density in the belt webbings.

Typical strain-resistance curves of webbings in the stretch-recovery cycles are shown in Figure 5. Each curve was plotted using the average of nine measurements. The results show the linear resistance response to the strain in the stretch-recovery measurement within 30% strain for all the webbings, in which the coefficients of determination (R^2^) of the linear regression curves was 0.99. The electrical resistance (*R*) of the materials is equal to $R=\rho \frac{L}{A}$. Here, *ρ* is resistivity, *L* is measured length, and *A* is cross section of the sample. In this work, when the webbing was stretched, the conductive yarns which were overfed in the relaxed state in the webbing were extended. The resistivity (*ρ*) and the cross section (*A*) of the conductive fiber in the webbing are not changed. The resistance change of the webbing depended on the extended length of the conductive fiber in the webbing. If the strain in the webbing does not surpass the feed ratio of the conductive fiber in the webbing, the linear resistance responding to the strain in the stretch-recovery measurement can be obtained. No electrical hysteresis was found in this work. The resistance sensitivity of the webbings in the stretch-recovery measurements within 30% strain was calculated. In this study the resistance sensitivity of the webbings is defined as the resistance change of the webbing under strain (%). The results are shown in Table 3. The resistance sensitivity of the webbings was affected by the number and the feed ratio of the conductive yarns, and the contact resistance of the webbings. The measured sensitivity of webbing (S_mw_) was calculated as follows: S_mw_ = ΔΩ_w_ ÷ 30%. Here, ΔΩ_w_ is the resistance change of webbing under strain (30%). The normalized sensitivity of webbing (S_nw_) was calculated as follows: S_nw_ = ΔΩ_w_ ÷ 30% × N_w_ ÷ L_w_. After normalizing the number of PAC fibers and the feed ratio of conductive yarn in the webbings, the normalized resistance sensitivity of the PAC fibers per 10 cm of FE08C85, TE08C80, and BE08C88-D04 were nearly the same. The differences in contact resistance with varied weft yarn densities in the belt webbings were found. The normalized resistance sensitivity of the PAC fibers per 10 cm of belt webbing increased in the order of BE12C88-D04, BE12C88-D08, and then BE12C88-D12. The more the weft yarn in the belt webbing, the higher the normalized resistance sensitivity of the PAC fiber. The influence of the number and the feed ratio of the conductive yarns and the contact resistance of the webbings on the resistance sensitivity of the webbings are obvious.

## 4. Conclusions

The strain-resistance in the stretch-recovery measurement of elastic-conductive webbings including flat, tubular, and belt webbings made by Lycra fibers and carbon coated polyamide fibers were investigated. The results showed that all the webbings had a good linear elasticity relationship between tensile load and strain when stretched, and when the recovery curves were within 30% strain. However, our findings show that there is tensile hysteresis in the webbings during the stretch-recovery measurement. When used as a textile strain sensor, this tensile hysteresis in the stretch-recovery cycle is detrimental to the durability of the strain sensor. The loss of tensile hysteresis of the webbing is due to the hysteresis of the Lycra fibers themselves, the friction among the yarns, and the webbing structure including the yarn density of the belt webbing in the weft. The decrease of friction among the yarns in the webbings decreases the loss of tensile hysteresis. It was found that compared with flat and tubular webbings, belt webbing constructed by two separate layers shows less loss of tensile hysteresis.

The strain-resistance curves of the webbings in the stretch-recovery measurements show that all the webbings show a good linear relationship between resistance and strain. No electrical hysteresis was found in any of the webbings. The resistance sensitivity of the webbing is affected by the conductivity of the PAC fibers, the number and the feed ratio of the conductive yarns, and the contact resistance of the webbing. The contact resistance of the webbing is not only influenced by the number of the conductive yarn, but also by the webbing structure and the weft yarn density in belt webbing. The results show that flat and tubular webbings have a lower ratio of contact resistance to measured resistance.

The stability of elasticity and the resistance sensitivity are two important properties for e-textiles when used as a strain-resistance sensor. In this study, it was found that the tensile hysteresis and the contact resistance influence the elasticity and the resistance sensitivity of the elastic-conductive webbings, respectively. The lower friction among the conductive yarns in the webbing contributes to the lower loss of tensile hysteresis. The closer the conductive yarns in the webbing, the lower the contact resistance. The balance between the loss of tensile hysteresis and contact resistance should be considered in the structural design of webbing as a strain-resistance sensor. The tensile hysteresis and the electrical conductivity of the webbings are affected by the measurements as well. They will be investigated in our further study to refine the performance of the textile strain sensor.

## Figures and Tables

**Table 1.** The number of elastic yarns and conductive yarns, the density of the weft yarns, and the feed ratio of the conductive yarns of the samples.

**Sample Code**	**Webbing Structure**	**Number of Elastic Yarns**	**Number of Conductive Yarns**	**Density of Weft Yearns (number/cm)**	**Feed Ratio (%)**
FE08C85	Flat	8	85		370
FE16C85	Flat	16	85		340
TE08C80	Tubular	8	80		330
TE16C80	Tubular	16	80		325
BE08C56-D04	Belt	8	56	4	310
BE12C88-D04	Belt	12	88	4	280
BE12C88-D08	Belt	12	88	8	255
BE12C88-D12	Belt	12	88	12	185
BE16C120-D04	Belt	16	120	4	315

Notes: F,T and B in the sample code indicate the flat, tubular and belt webbings, respectively; E08 indicates the eight elastic yarns; C85 indicates the eight-five conductive yarns; D04 indicates that the density of the weft yarns is four threads per cm.

**Table 2.** Hysteresis loss of webbings during the stretch-recovery measurements at 30% strain.

**Number of Lycra Fibers**	**Hysteresis Loss**				
	**Lycra Fibers (N·mm)**	**Elastic Yarn (N·mm)**	**Webbing**		
			**Flat (N·mm)**	**Tubular (N·mm)**	**Belt (N·mm)**
8	13.4 ± 1.2	15.8 ± 1.5	22.3 ± 1.6	24.4 ± 1.3	17.6 ± 0.7

					28.4 ± 0.7 (BE12C88-D04)
12	18.6 ± 0.4	23.5 ± 0.8			31.1 ± 1.4 (BE12C88-D08)
					55.5 ± 1.6 (BE12C88-D12)

16	22.6 ± 1.1	26.3 ± 0.9	42.1 ± 0.7	43.8 ± 1.0	34.7 ± 0.7

**Table 3.** Resistance and sensitivity of samples within a 30% strain.

**Sample Code**	**Measured Resistance (kΩ/10 cm)**	**Normalized Resistance of PAC Fiber (kΩ/10 cm)**	**Measured Sensitivity of Webbing (ΔΩ/%)**	**Normalized Sensitivity of PAC Fiber (ΔΩ/%)**
PAC Fiber	4,077 ± 255			
Conductive yarn	329 ± 3	4,941 ± 41		
FE08C85	14.9 ± 0.2	5,136 ± 57	103.6 ± 5.7	35.7 ± 1.6
TE08C80	14.2 ± 0.1	5,152 ± 30	98.9 ± 1.9	36.0 ± 0.7
BE12C88-D04	11.7 ± 0.1	5,526 ± 30	72.7 ± 2.8	35.1 ± 0.5
BE12C88-D08	10.8 ± 0.1	5,601 ± 33	74.4 ± 2.0	38.5 ± 1.0
BE12C88-D12	8.3 ± 0.1	5,944 ± 37	60.0 ± 1.4	42.8 ± 1.0

**Table 4.** Contact resistance of the conductive yarn and webbings.

**Sample Code**	**R_i_ + R_c_ of Sample (kΩ/10 cm)**	**Normalized R_c_ of PAC Fiber (kΩ/10 cm)**
PAC Fiber	4,077 + 0	0
Conductive Yarn	272 + 57	855
FE08C85	11.8 + 3.1	1,068
TE08C80	11.2 + 3.0	1,091
BE12C88-D04	8.6 + 3.1	1,461
BE12C88-D08	7.9 + 2.9	1,501
BE12C88-D12	5.7 + 2.6	1,855
